# The Choice of Peritoneal Dialysis Catheter Implantation Technique by Nephrologists

**DOI:** 10.1155/2013/940106

**Published:** 2013-01-28

**Authors:** T. Yip, S. L. Lui, W. K. Lo

**Affiliations:** Dr. Lee Iu Cheung Memorial Renal Research Centre, Tung Wah Hospital, Department of Medicine, The University of Hong Kong, Hong Kong

## Abstract

Peritoneal dialysis catheter (PDC) is the lifeline of peritoneal dialysis (PD) patients. One of the critical issues for successful PD is a well-functioning PDC which is timely inserted. It is the implantation technique rather than the catheter design that determines the outcome of the catheter. Dedication in acquiring the appropriate technique is vital to the success of a PD program. In this paper, we discuss the pros and cons of various techniques used for PDC implantation. A detailed description of PDC implantation by using the minilaparotomy method is presented. We strongly recommend mini-laparotomy as the method of choice for PDC implantation by nephrologists.

Peritoneal dialysis (PD) is a well-established technique of renal replacement therapy in patients with end-stage renal disease (ESRD). The advantages of PD include preservation of residual renal function, better patient survival in the first few years, better quality of life and cost-effectiveness over hemodialysis [1–4]. Thus, PD is well suited to act as a first-line renal replacement therapy in an integrated approach to end-stage renal failure care. In Hong Kong, “PD-first” policy has been adopted since mid-1980s. Currently, up to 80% of ESRD patients on maintenance dialysis are on PD. It has provided a successful model for the PD first policy.

 For a PD program to succeed, access to peritoneal dialysis catheter (PDC) implantation must be timely and the procedure must be performed by an experienced operator with low catheter failure rates and complications. PDC can be implanted percutaneously or by open surgery (Table 1). The standard percutaneous placement includes the “trocar and cannula” method and the Seldinger technique, with variations like fluoroscopy-assisted or peritoneoscopy-assisted placement. Open surgical approach includes minilaparotomy and laparoscopic placement.

In many centers, PDC is implanted by surgeons, either by minilaparotomy or laparoscopic approach. However, referral to surgeons usually causes delay in initiating PD therapy, for both the waiting time to see a surgeon and the time required to arrange the operation afterwards. The date of implantation is often not under the control of nephrologists and this may make timely implantation of a PDC an impossible dream. Some patients may be forced to remain on hemodialysis with a central venous catheter, which is associated with an accelerated decrease in residual renal function and high rates of bacteremia and mortality. Survival data from the United States consistently showed a higher mortality in patients started with hemodialysis in the first three months [5–7]. Together with the large PD patient load, nephrologists in Hong Kong are obliged to insert PDCs by themselves. In fact, the success of PD access procedures performed by nephrologists using various techniques has been well documented [8–12]. Catheter insertion by nephrologists has been shown to improve PD utilization and increase the PD population growth rate in other parts of the world [9, 11, 13, 14].

Conventionally, nephrologists prefer the percutaneous approach. The percutaneous techniques are relatively simple to perform. They require a short learning period and can be performed in a clean side-room under local anesthesia. The “trocar and cannula” technique is the first method adopted by nephrologists for the implantation of PDCs. With this technique, the trocar's sharp pointed stylet is pushed through the linea alba into the lower abdomen. After entry into the peritoneal cavity, the stylet is removed and the PDC is passed with a stiffening stylet into the peritoneal cavity toward the pelvis. The side pieces of the trocar are removed with the internal cuff of the catheter situated above the linea alba. This technique is easy to perform but due to its blind entry into the peritoneal cavity with the sharp and thick trocar, complications are common. Serious complications, such as perforation of the bladder or bowel, jejunal mesenteric artery laceration, and even laceration of the spleen, have been reported [15–17]. Risk of subsequent incisional hernia development is high [17]. In our opinion, the trocar and cannula method should not be used for PDC insertion because the blind insertion of the sharp and thick trocar is inherently associated with the risk of viscera perforation or damage, no matter how careful and experienced the operator is [18]. The Seldinger technique was developed to improve the results of bedside PDC implantation. With the Seldinger approach, a guidewire is inserted through a priming needle. An introducer, dilators, and a peel-away sheath are then inserted along the guidewire. The guidewire is removed and the PDC is inserted with a stiffening stylet through the sheath, as in the trocar and cannula technique. The peel-away sheath is then separated and removed. The subcutaneous tunnel is created in the usual way. It requires priming the peritoneal cavity with 2 liters of peritoneal dialysate to prevent visceral injury from the blind puncture of the priming needle. In contrast to the trocar and cannula technique, this technique is less traumatic. Although it is also a blind procedure, reported complication rates are much lower compared to the trocar and cannula technique [12, 19]. In some centers using the Seldinger technique, the catheter survival rates were even better than that implanted by open surgical method in selected groups of patients without prior abdominal surgery [20, 21]. In the recent years, there has been an increase in the utilization of peritoneoscopic implantation of PDC with the Y-TEC system by nephrologists. This method adopts the Seldinger technique for catheter placement but allows direct visualization of the peritoneal cavity after air insufflation, thus avoiding placing the catheter under bowel loops, omentum, or against adhesions. Good results have been reported [8, 22]. Although peritoneoscopic implantation of PDC provides direct visualization, the introduction of the peritoneoscope still involves blind insertion through the abdominal wall. Therefore, bowel perforation remains a potential serious complication [13]. Pneumoperitoneum and pneumomediastinum are the rare complications caused by the air insufflation. The major limitation of this approach is the high cost of the peritoneoscope system and the disposable consumables.

The safety of the percutaneous technique is further improved by using ultrasound and fluoroscopic guidance. Ultrasound helps the operator to identify and avoid damaging blood vessels like the inferior epigastric artery and vein, during abdominal puncture. In the fluoroscopic guided insertion, contrast may be used after the puncture needle has entered the peritoneum to ensure that the needle has not entered the bowel. The guidewire and the PDC can be visualized during the advancement into the pelvis. However, the danger of bowel perforation and organ damage still exists because the peritoneum is not punctured and entered under direct visualization and this is the main limitation of all percutaneous methods. The percutaneous approach is, therefore, relatively contraindicated in patients with previous abdominal surgery or peritonitis. Furthermore, the peritoneum opening cannot be sutured, causing a relatively high incidence of early leakage. Pericatheter leaks predispose to catheter exit site infection and peritonitis. Persistent leak calls for catheter removal. Reported early leakage rates range from 5% to 20% [21, 23–26]. To decrease the incidence of leakage, it is recommended to have a break-in period of 7 to 14 days for commencement of PD [27, 28]. However, patients often are reluctant to start dialysis without uremic symptoms, and the onset of uremic symptoms is relatively sudden among those with low glomerular filtration rate. To allow immediate PD after catheter implantation with low early leakage rates and other complications, open surgical implantation by minilaparotomy is the best solution.

Nephrologists can be trained to perform open surgery with minilaparotomy for PDC implantation. We have been using surgical approach by minilaparotomy for PDC insertion in our center for more than 20 years. Several nephrologists have been trained to perform the procedure competently. Patients with previous uncomplicated abdominal operations other than colectomy like hysterectomy and cholecystectomy are not excluded. Preoperatively, the beltline of the patient is identified in the standing and sitting positions. The main wound, the tunnel, and the exit site are marked in such a way that the position of the future exit site is few centimeter away from the beltline. The location of the main wound is around 10 cm above the pubic symphysis and 2 cm lateral to the midline (Figure 1). We usually use the conventional straight Tenckhoff catheter which is 42 cm in length. Longer catheter is chosen when the positions of the main wound and the exit site are higher. Patients are asked to empty their bladder before the procedure. We routinely performed bladder ultrasound scanning to exclude urinary retention after voiding. This prevents perforation of the urinary bladder during catheter insertion [29–31]. Prophylactic antibiotic is routinely given. The procedure is performed in a day-care operating room under local anesthesia without anesthetist support. 2% lignocaine is used as a local anesthetic agent, with intravenous midazolam when needed. After paramedian skin incision, the subcutaneous tissue is dissected till the reach of the anterior rectus sheath. Paramedian placement reduces the risk of pericatheter leak and hernia and enhances tissue ingrowth into the deep cuff with firm fixation of the catheter [32, 33]. The anterior rectus sheath is then opened, and the rectus muscle is bluntly split. The posterior rectus sheath and the peritoneum are then identified and cut open. Purse-string suture of the peritoneum together with the posterior rectus sheath is then applied. After the insertion of PDC with a malleable stylet, the peritoneum and posterior rectus sheath are closed with the purse-string suture. The internal cuff is tightly tied above the posterior rectus sheath and the peritoneum, secured within the rectus muscle. Free drainage is tested, followed by indwelling of PD fluid to test for pericatheter leakage. The tight purse string suture and direct visualization for leakage check greatly reduce the chance of leakage even with immediate commencement of PD. The anterior rectus sheath is then sutured with a part of the PDC tunneled between the anterior rectus sheath and the rectus muscle. This rectus sheath tunneling is an important adjunctive technique to keep the catheter positioned in the pelvis and to prevent catheter tip migration [34–36]. After the PDC is tunneled in the subcutaneous layer, a downward pointing exit site is created with the external cuff located at 2 cm from the exit site. No suture is placed at the skin exit. The wound and the exit site are covered with nonocclusive dressings. Adhesives are applied to fix the PDC on the abdominal wall. In our center, intermittent PD is performed in almost all patients immediately after implantation, and yet leakage is almost absent and catheter malfunction from other causes is also very uncommon [10, 37]. Excellent PDC outcomes have also been reported from other centers using minilaparotomy as the insertion technique by nephrologist [38–40]. To many nephrology services, the requirement of an operating room setting for minilaparotomy may be a limiting factor. However, in Hong Kong, it is still safely implanted in operating rooms converted from sidewards.

Compared to the percutaneous techniques, the learning time for minilaparotomy is longer. Apart from mastering the surgical steps, the nephrologist has to learn to handle potential intra-operative complications such as arterial bleeding. But once the skill is acquired, the nephrologist will find the satisfaction of freedom from postoperative troubles of blind implantations, and will have the control of arranging the catheter implantation procedures. Other PDC-related procedures including PDC removal, exteriorization and shaving of the external cuff, simultaneous removal, and reinsertion of PDC [41] are made possible after acquiring minilaparotomy techniques.

Laparoscopy is increasingly being used as a modality for establishing peritoneal access and various laparoscopic techniques have been described for catheter placement. Laparoscopy provides the ability to directly visualize placement of the catheter tip in pelvis and proactively address anatomic problems that may result in mechanical catheter dysfunction. Lysis of adhesions, omentopexy, peritoneal biopsy, and hernia repairs can be done at the time of catheter placement. However, laparoscopy requires longer duration of operation and is more costly than open surgical insertion because specialized equipment is required. Laparoscopy has an inherently steep learning curve. Experienced surgeons are required. In addition, general anesthesia is usually required and many ESRD patients are high-risk candidates for general anesthesia for their multiple comorbid conditions. Thus, laparoscopy approach should be reserved for the needy patients like those who are expected to have intra-abdominal adhesions rather as a routine first-line approach.

PDC is the lifeline of PD patients. One of the critical issues for successful PD is a well-functioning PDC which is timely inserted. PDC insertion must be regarded as an important procedure, demanding care and attention to detail. There is no PDC that is definitely better than the conventional double-cuffed Tenckhoff catheter [42, 43]. It is the implantation technique rather than the catheter design that determines the outcome of the catheter. Dedication in acquiring the appropriate technique is vital to the success of a PD program. We strongly recommend minilaparotomy as the method of choice for PDC insertion by a nephrologist providing that an operating room setting is available. The percutaneous approach by the Seldinger technique is a good alternative method of PDC implantation in selected patients without prior abdominal surgery. Most importantly, the operators should be well trained for the technique chosen and the outcome monitored regularly.

## Figures and Tables

**Figure 1:**
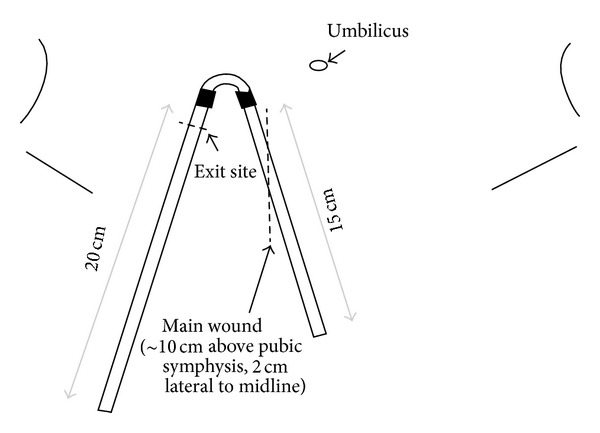
Location of main wound and exit site.

**Table 1:** Comparison of different methods of peritoneal dialysis catheter implantation.

Method	Trocar and cannula	Seldinger technique	Minilaparotomy	Laparoscopic
Done by	Nephrologist	Nephrologist	Nephrologist/surgeon	Surgeon
Setting	Clean side-room	Clean side-room	Operating theater	Operating theater
Anesthesia	Local anesthesia	Local anesthesia	Local/general anesthesia	General anesthesia
Pros	Short learning time	(i) Short learning time (ii) Low complication rates	(i) Direct visualization of peritoneum (ii) Allow purse-string suture of peritoneum (iii) Low leakage rates	(i) Visualization of intra-abdominal structures (ii) Allow adjunctive procedures for example Adhesiolysis, omentopexy
Cons	High complication rates	Relatively high early leakage rates	Long learning time	(i) Very long learning time (ii) High cost (iii) Specialized equipment needed
